# A Rare Case Report of Limb Body Wall Complex

**DOI:** 10.7759/cureus.59026

**Published:** 2024-04-25

**Authors:** Ruchika Narayan, Anamika Meena, Rajib Sarkar, Muskaan Agrawal

**Affiliations:** 1 Radiodiagnosis, All India Institute of Medical Sciences, Patna, IND

**Keywords:** limb deformity, congenital anomaly, body stalk syndrome, gastroschisis, limb body wall complex

## Abstract

Limb body wall complex (LBWC), also known as body stalk anomaly, is a rare and lethal disorder of the anterior abdominal wall. It is characterized by a severe combination of congenital malformations in the fetus, including, abdomino- and/or thoracoschisis, exencephaly/encephalocele, limb deformities, and facial clefts. Short umbilical cord, abdominal placental attachment, and spinal anomalies are among other manifestations of this disorder. The cause of LBWC is still unknown. The main hypotheses include embryonic dysplasia, early amniotic rupture, and vascular accident during embryonic development. We present a case of LBWC that was detected prenatally on ultrasound (USG) imaging and later confirmed postnatally in a Rh-negative mother at the menstrual age of 14 weeks.

## Introduction

Limb body wall complex (LBWC) is a rare congenital and sporadic fetal condition consisting of many disruptive abnormalities [1-5]. Because the majority of afflicted fetuses die in utero, the incidence of LBWC at birth is approximately 0.32 per 100,000 births [1-4]. LBWC is also known as body stalk syndrome. Van Allen et al. have provided a more detailed description of this condition, and based on their criteria, the diagnosis of LBWC can be established based on any two of the following features: 1) encephalocele/encephaly with clefts in the face, 2) thoraco- and/or abdominoschisis, and 3) deformity of the limbs [6]. Given that it is regarded as a fatal defect, prompt prenatal diagnosis becomes crucial in order to provide parents with advice for timely pregnancy termination [1]. With its distinctive features, prenatal ultrasonography can be helpful in obtaining an early diagnosis of this condition. We report here a prenatal ultrasound (USG)-based diagnosis of LBWC that was made at 14 weeks following the start of amenorrhea and confirmed after delivery.

## Case presentation

A 19-year-old, gravida two, para one, woman with 14 weeks of gestation, presented with a history of bleeding per vagina for two days. She was referred to the radiodiagnosis department for fetal ultrasonography. The woman tested negative for the Rh factor, although her husband was Rh positive. She also had a previous history of spontaneous abortion at seven months gestation age. Even though the fetus's Rh status was unknown, the mother did receive prophylactic anti-D immunoglobulin. There was no history of inherited diseases or deformities in the family. Furthermore, there was no history of any medical illness or surgery, nor any history of using addictive substances or potentially harmful medications. The current pregnancy was conceived spontaneously. There was no history of a prior fetal scan and folic acid supplement. The patient was mildly anemic (Hb: 9.5g/dL); however, the rest of the laboratory investigations were unremarkable.

**Figure 1:**
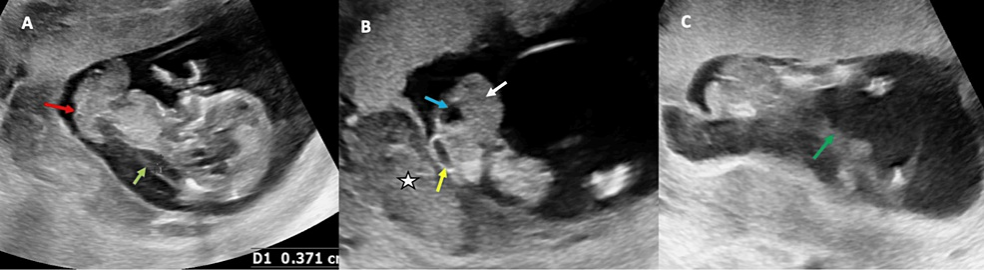
USG image showing abdominal wall defect with evisceration of abdominal organs (red arrow) and an increased nuchal translucency (light green arrow). A,B: the eviscerated organs included liver (white arrow), echogenic clumped bowel loops with stomach (yellow arrow), and urinary bladder (blue arrow); B: sub-chorionic hemorrhage (asterisk); C: hypoplastic right lower limb (green arrow). USG, ultrasound

On ultrasonography, the crown-rump length was shorter; however, the head circumference, biparietal diameter, and femur length were in line with menstrual age (approximately 14 weeks). The gestational age (GA) according to USG was two weeks behind the GA according to the last menstrual period. In addition, a large abdominal wall defect was present, along with evisceration of the abdominal organs without any visualized protective membrane that covers the umbilical cord. The identified eviscerated organs included the liver, echogenic clumped bowel loops with the stomach, and urinary bladder (Figure 1). The thickness of the nuchal translucency was increased (>3.5 mm). 

**Figure 2:**
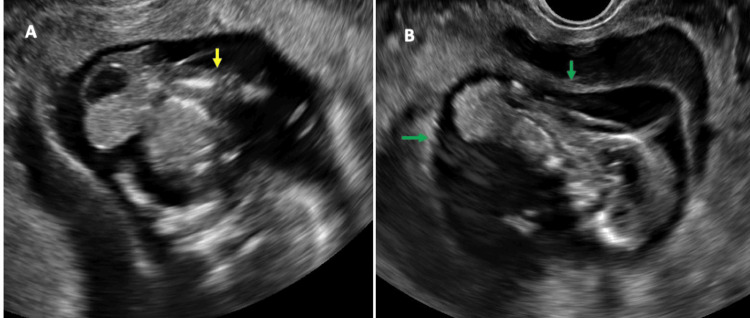
USG image showing eviscerated organs with a deformed left lower limb (yellow arrow) (A), and a wide chorioamniotic separation (green arrow) (B). USG, ultrasound

Although the three segments of the left lower limb and both upper limbs were visible, the left lower limb appeared malrotated with a deformed foot, which raised the probability of club foot (Figure 2). The three bony segments of the right lower limb were not visible, only a small, oblong limb-like soft tissue was found. The cranial structures were normal for age. Additionally, observed was subchorionic hemorrhage with a wide gap between the chorion and amnion (Figures 1, 2). A very short portion of the umbilical cord was visible. The intact amniotic membrane along with the eviscerated organ was found to be focally adhered to the placenta. Amniotic fluid was adequate. Overall, USG findings were consistent with the diagnosis of LBWC.

**Figure 3:**
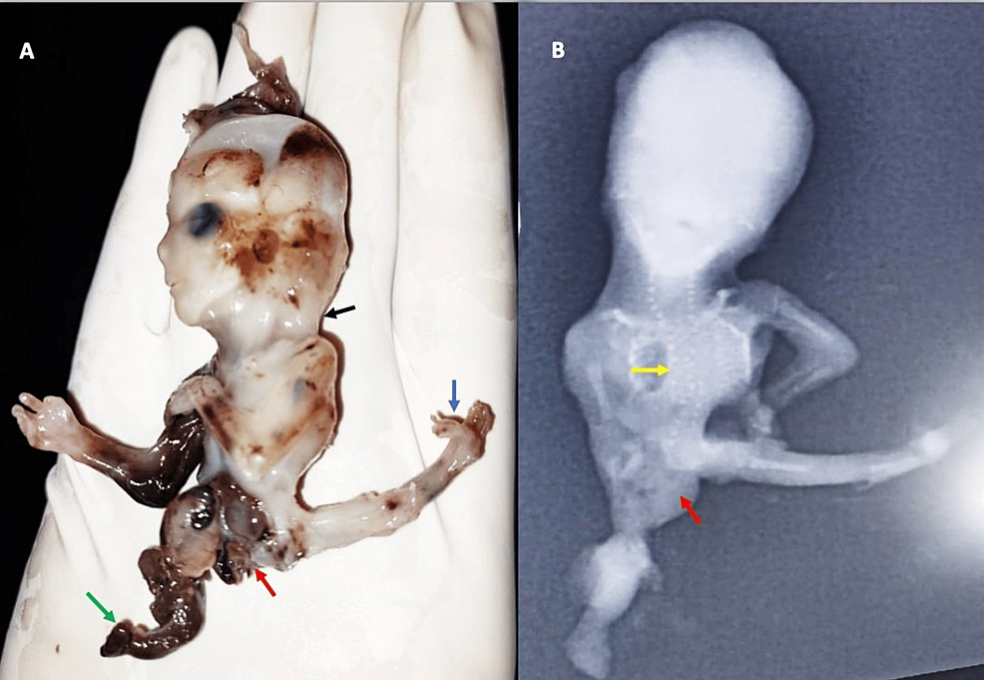
Post-delivery image (A) showing eviscerated abdominal organs without membranous covering (red arrow), nuchal thickening (black arrow), a limb-like soft tissue adherent to the externalized abdominal organs on the right side, hypoplastic right lower limb (green arrow), and a malrotated left lower limb with deformed foot (blue arrow). On post-natal X-ray imaging (B), distorted abdominal contour was noted with ill-defined opacities. Soft tissue opacification (red arrow) was seen along the right lower limb; however, no defined ossified bones in any of the three segments were noted compared to the left side. Furthermore, a mild kyphoscoliotic curvature of the spine with unossified vertebral bodies in the cervical and lumbosacral region (yellow arrow) was also noted.

The parents were informed about the state of the fetus and its dismal prognosis. Subsequently, the dead fetus was spontaneously evacuated through the vagina. The malformed foot, malrotated left lower limb, and eviscerated organs were all visible, along with the hypoplastic right lower limb (Figure 3). Thus, the USG findings were validated. Additional observations on the infantogram were the absence of the right lower limb bones, an abnormally curved spine, and an unossified centrum with splaying neural arches in the cervical and lumbosacral regions (Figure 3). The parents' karyotypes were found to be normal, but they declined to have their fetus karyotyped.

## Discussion

The first description of LBWC was made in 1987 by Van Allen et al. [6]. It is an extremely rare and fatal disorder of the anterior abdominal wall. The three main characteristics of this disorder are as follows: first, the thoracic and/or abdominal walls are severely disrupted; second, the organs (liver, bowel, bladder, heart, etc.) are located outside the body cavity and are contained within an amniotic sac that is closely attached to the placenta, resulting in a relatively fixed position for the fetus; and third, the umbilical cord is either very short or nonexistent, possibly due to improper development of the body stalk.

Associated musculoskeletal abnormalities, such as clubfoot and nonexistent or deformed limbs, and aberrant curvature of the spine (kyphoscoliosis) are frequently observed in LBWC. Our case presented similar findings with a substantial disruption of the abdominal wall, eviscerated organs, and abnormalities in the spine and limbs. Several criteria have been proposed for the diagnosis of LBWC syndrome. The Van Allen classification is the easiest to use. It consists of three features, two of which are necessary for diagnosis [6]. The criteria proposed by them to diagnose include exencephaly/encephalocele with a facial cleft, thoraco/abdominoschisis, and limb defects. The last two features were present in our case.

Although the exact etiopathogenesis of this condition is still unknown, a number of theories have been put forth, such as early vascular disruption, the disruption brought on by the formation of amniotic bands after early amniotic rupture, or embryonic dysplasia as a result of the embryonic disc's folding process completely failing. The most well-recognized explanation is that of embryonic dysplasia [6-8]. Our case study might support the early vascular disruption hypothesis by Allen et al. as there is a probability of Rh incompatibility-associated isoimmunization.

There is a recurrence risk of this condition as observed in the two families so far, which may indicate a potential genetic predisposition [9]. There is no known treatment for this illness, and it is invariably fatal. It is crucial to distinguish this disorder from other anterior abdominal wall abnormalities, such as isolated gastrochisis and omphalocele, as these have better prognoses than LBWC [10-12]. Prenatal USG can detect this anomaly as early as the first trimester with its common sonographic appearances like exencephaly/encephalocele with a facial cleft, thoraco- and/or abdominoschisis, limb defect, scoliosis, and small/absent umbilical cord. Increased maternal serum alpha-fetoprotein levels can add to the diagnosis of this condition.

## Conclusions

LBWC is a rare fetal disorder characterized by multiple congenital anomalies, including exencephaly/encephalocele, thoraco- and/or abdominoschisis, and limb defects. Early antenatal diagnosis of this condition is possible by USG examination. Because this condition is usually fatal, it must be distinguished from gastroschisis, which has a better prognosis.
